# Characterization and mapping of leaf rust resistance in four durum wheat cultivars

**DOI:** 10.1371/journal.pone.0197317

**Published:** 2018-05-10

**Authors:** Dhouha Kthiri, Alexander Loladze, P. R. MacLachlan, Amidou N’Diaye, Sean Walkowiak, Kirby Nilsen, Susanne Dreisigacker, Karim Ammar, Curtis J. Pozniak

**Affiliations:** 1 Department of Plant Sciences, Crop Development Centre, University of Saskatchewan, Saskatoon, Saskatchewan, Canada; 2 International Maize and Wheat Improvement Center (CIMMYT), Mexico, D.F., Mexico; Institute of Genetics and Developmental Biology Chinese Academy of Sciences, CHINA

## Abstract

Widening the genetic basis of leaf rust resistance is a primary objective of the global durum wheat breeding effort at the International Wheat and Maize Improvement Center (CIMMYT). Breeding programs in North America are following suit, especially after the emergence of new races of *Puccinia triticina* such as BBG/BP and BBBQD in Mexico and the United States, respectively. This study was conducted to characterize and map previously undescribed genes for leaf rust resistance in durum wheat and to develop reliable molecular markers for marker-assisted breeding. Four recombinant inbred line (RIL) mapping populations derived from the resistance sources Amria, Byblos, Geromtel_3 and Tunsyr_2, which were crossed to the susceptible line ATRED #2, were evaluated for their reaction to the Mexican race BBG/BP of *P*. *triticina*. Genetic analyses of host reactions indicated that leaf rust resistance in these genotypes was based on major seedling resistance genes. Allelism tests among resistant parents supported that Amria and Byblos carried allelic or closely linked genes. The resistance in Geromtel_3 and Tunsyr_2 also appeared to be allelic. Bulked segregant analysis using the Infinium iSelect 90K single nucleotide polymorphism (SNP) array identified two genomic regions for leaf rust resistance; one on chromosome 6BS for Geromtel_3 and Tunsyr_2 and the other on chromosome 7BL for Amria and Byblos. Polymorphic SNPs identified within these regions were converted to kompetitive allele-specific PCR (KASP) assays and used to genotype the RIL populations. KASP markers *usw215* and *usw218* were the closest to the resistance genes in Geromtel_3 and Tunsyr_2, while *usw260* was closely linked to the resistance genes in Amria and Byblos. DNA sequences associated with these SNP markers were anchored to the wild emmer wheat (WEW) reference sequence, which identified several candidate resistance genes. The molecular markers reported herein will be useful to effectively pyramid these resistance genes with other previously marked genes into adapted, elite durum wheat genotypes.

## Introduction

Durum wheat (*Triticum turgidum* L. ssp. *durum*) is a widely grown crop used in the preparation of diverse food products including bread, couscous, bulgur and pasta. As the main global provider of improved wheat germplasm, the International Wheat and Maize Improvement Center (CIMMYT) sustains a breeding effort addressing all issues important to the viability of durum wheat crops worldwide. Canada is among the world’s largest producers and is the largest exporter of durum wheat. Canada also maintains an extensive genetic improvement effort of durum wheat, aiming at enhancing the competitiveness of this crop for Canadian farmers. Leaf rust, caused by the fungal pathogen *Puccinia triticina* Eriks, is a major biotic constraint threatening the productivity of durum wheat worldwide, thereby representing an important breeding objective for both programs. This foliar disease is capable of causing considerable grain yield losses and quality downgrades [1,2]. Improvement of resistance is the most cost-effective and environmentally viable strategy for controlling leaf rust, and deployment of cultivars with durable resistance is a major target of wheat breeding programs globally [3–5].

Over 76 genes conferring resistance to leaf rust (*Lr* genes) have so far been identified and localized to specific wheat chromosomes. Most of these genes originated from hexaploid bread wheat (*T*. *aestivum* L.) or wild grass species related to wheat, while a limited number have been found and characterized in tetraploid durum wheat [6]. In both durum and bread wheat, monogenically inherited *Lr* genes have usually been defeated by new, rapidly evolving races of *P*. *triticina* with different virulence patterns [2,7]. Durum wheat has historically been more resistant to leaf rust than bread wheat [8,9], and most of the predominant *P*. *triticina* isolates found on common wheat are avirulent on a large number of durum wheat genotypes [10–12]. However, with the appearance of more durum-specific races of the pathogen, and the breakdown of resistance in several countries during the last decade [2,13,14], leaf rust has become a primary challenge for durum breeders globally. The detection and spread of the new *P*. *triticina* race BBG/BN with virulence to *Lr72*, a widely-deployed gene in the CIMMYT durum germplasm, has led to severe epidemics in northwestern Mexico, from 2001 to 2003 [8,15]. Since then, genetic studies conducted at CIMMYT have led to the identification of effective resistance genes in modern durum wheat germplasm, including the linked genes *Lr3* and *Lr*_*Camayo*_, both mapped to chromosome 6BL [16], the complementary gene pair *Lr27+31*, located on chromosome arms 3BS and 4BS, respectively [17], *Lr14a* on chromosome 7BL [18] and the newly designated *Lr61* on chromosome 6BS [19]. As the race BBG/BN continued to evolve, a new variant identified as BBG/BP acquired virulence to the complementary resistance genes *Lr27+Lr31* in 2008 [2]. Race BBBQD, with a similar virulence pattern to the Mexican races of *P*. *triticina*, was detected in durum fields in California, USA, during 2009 [20]. In 2013, this highly virulent race was reported in Kansas, USA, increasing the risk of its spread northward to the major durum-producing areas of North Dakota, USA and Saskatchewan, Canada [21,22].

Diversification and widening of the genetic basis for leaf rust resistance in durum wheat, and breeding for durable resistance, are both critical for the sustainability of its production. The recent revolution in next generation sequencing technologies [23–25] and the development of low-cost and high-throughput SNP genotyping systems [26–29] have promoted the rapid development of reliable markers for marker-assisted breeding in wheat, while providing efficient tools for mapping resistance genes.

Selective genotyping [30] and pooled DNA analysis or bulked segregant analysis (BSA) [31,32] are two cost-saving, yet effective approaches to rapidly identify candidate regions for genes of interest, by genotyping selected individuals or pooled DNA samples from the high and low tails of the phenotypic distribution of a population. Linkage between the phenotype and the markers is then inferred by analyzing allele frequencies between the groups of individuals or bulks with contrasting phenotypes. Both approaches have been used to map rust resistance genes in wheat [19,33,34]. The objectives of the present study were to (1) characterize the genetic basis of leaf rust resistance in the four durum genotypes Amria, Byblos, Geromtel_3 and Tunsyr_2, which express resistance to all currently known races of *P*. *triticina* in Mexico, and (2) to develop tightly linked molecular markers that would be useful for marker-assisted breeding and gene pyramiding.

## Material and methods

### Plant materials

Four RIL populations were developed by crossing the highly susceptible CIMMYT line ATRED #2 (pedigree: Atil*2/LocalRed) to four resistance sources, namely, Geromtel_3 and Tunsyr_2 from the International Center for Agricultural Research in the Dry Areas (ICARDA) (pedigrees: Gersabil_1/4/D68.1.93A.1A//Ruff/Flamingo/3/Omtel_5 and D68.1.93A.1A//Ruff/Flamingo/3/Omtel_5/4/Lahn, respectively), Amria, from Morocco (pedigree: HadjMouline/Saada//Karim), and Byblos, a French durum cultivar of unknown pedigree. These sources of resistance were selected based on information generated by CIMMYT’s durum wheat breeding team, for their seedling and adult plant resistance at several locations and over multiple cropping cycles, in Mexico and worldwide [35]. Crosses and generation advancement were made at CIMMYT’s experimental stations in Mexico, as described in detail in Loladze et al. [35].

### Field experiments and phenotyping

Three generations of the RILs and the parental genotypes were evaluated for the disease response in the field, in two different environments in Mexico. During the summer of 2011, the F_2_-derived F_3_ (F_2:3_) families from each cross were space planted in double 1.2-meter-long rows in the CIMMYT field leaf rust nurseries at the El Batán experimental station, which allowed us to observe approximately 20 to 30 individual plants per family. The El Batán experimental station is located at CIMMYT headquarters near Mexico City (latitude 19.53, longitude -98.84, altitude 2250 m asl), where wheat is sown in mid-May and harvested in mid-October. In 2013, the F_2:6_ RILs were grown at the CENEB station in Ciudad Obregon, situated in the State of Sonora (latitude 27.33, longitude -109.93, altitude 35 m asl) in Northwestern Mexico, with a wheat crop season from mid-November to late April. Finally, the F_8_ RILs were phenotyped during the summer of 2014, at the field leaf rust nurseries in El Batán station. Field plots for the 2013 and 2014 trials were grown in 1.2-meter-long rows, with two replicates (paired rows) for each line and approximately 30 plants per replicate. ​Parental genotypes and susceptible and resistant checks were included in all field evaluations. A mixture of the susceptible cultivars Banamichi C2004 and Jupare C2001 (resistant to most Mexican races, except BBG/BP) was used as rust spreader rows. At the tillering stage, all plant materials were inoculated with race BBG/BP urediniospores suspended in light mineral oil (Soltrol 170), at a concentration of 5 to 10 mg of urediniospores per 5 ml of oil. The race BBG/BP of *P*. *triticina* was the predominant durum-specific race in Mexico, with the following avirulence/virulence formula: *Lr1*, *2a*, *2b*, *2c*, *3*, *3ka*, *3bg*, *9*, *13*, *14a*, *15*, *16*, *17*, *18*, *19*, *21*, *22a*, *24*, *25*, *26*, *28*, *29*, *30*, *32*, *35*, *37/Lr10*, *11*, *12*, *14b*, *20*, *23*, *27 + 31*, *33*, *72* [2,35].

The scoring of leaf rust reactions was performed at least twice during each growing season. The percentage of infected leaf area (disease severity) was estimated according to the modified Cobb scale [36]. Host reaction was also recorded using four categories: resistant (R) with miniature uredinia; moderately resistant (MR) as indicated by presence of small uredinia, moderately susceptible (MS) expressed as moderate sized uredinia and full susceptibility (S), with presence of many large uredinia [37]. In the F_3_ generation, depending on the host reactions of plants within a family, families were categorized as homozygous resistant (all plants resistant), homozygous susceptible (all plants susceptible) and segregating (plants originating from a heterozygous plant). The F_6_ and F_8_ RILs were scored as resistant (R) or susceptible (S), based on their host reaction. The chi-square (*χ*^*2*^) test was applied to determine the goodness of fit of the observed phenotypic distributions of the host reaction in the segregating populations to the expected genetic ratio for a monogenic inherited resistance.

### Allelism tests

Allelism tests were conducted as described in Loladze et al. [35], by screening F_2_ populations from crosses between the resistant parental lines for the presence of susceptible recombinants. A minimum of 181 and up to 304 F_2_ plants per cross were evaluated in these tests. Also, allelism to the known resistance gene *Lr61* was studied in F_2_ populations from crosses between the *Lr61*-carrying line Sooty_9/Rascon_37//Guayacan INIA and each of the four parental lines Amria, Byblos, Geromtel_3 and Tunsyr_2. In addition, in April 2013, 200 F_2_ plants generated from the cross Amria/Byblos were evaluated for their reaction to BBG/BP, at seedling stage in the greenhouse at CIMMYT. The infection types (ITs) of the resulting F_2_ seedling progenies were assessed using the 0 to 4 scale described by McIntosh et al. [38], where “0” = no visible leaf rust symptoms; “;” = hypersensitive flecks without any uredinia; “1” = small uredinia surrounded by necrosis; “2” = small to medium uredinia surrounded by chlorosis or necrosis; “3” = medium-sized uredinia with or without chlorosis; “4” = large uredinia without chlorosis or necrosis; “X” = random distribution of variable-sized uredinia, and “+” and “-” were used when uredinia were somewhat larger or smaller than the average for the IT class. ITs of 3, 3+ and 4 were considered to be susceptible host reactions, whereas all of the other ITs were considered resistant. In case of absence of susceptible recombinants in the F_2_ progenies, it was assumed that the two resistant parents carried allelic or closely linked leaf rust resistance genes.

### Bulked segregant analysis using the iSelect 90K SNP array

A bulked segregant analysis (BSA) approach was carried out on 15 resistant and 15 susceptible F_3_ families from each population, using the Illumina iSelect 90K Infinium SNP genotyping array [28]. Genomic DNA was extracted from the parental lines and the selected families using a modified CTAB method [39]. The quality of DNA was assessed on 2% agarose gel. DNA quantification was performed using PicoGreen fluorescence detection, and all DNA samples were diluted to 50 ng/μl. One resistant and one susceptible bulk DNA samples were created for each population, by pooling equal quantities of genomic DNA from the previously selected families. The parental lines, the selected families, and the bulks from each population were genotyped with the wheat 90K Infinium iSelect assay, using BeadStation and iScan, according to the manufacturer’s protocol from Illumina. SNP clustering and data analysis were performed using GenomeStudio software (Illumina, San Diego, CA, USA). Polymorphic SNPs that distinguished the parental lines and co-segregated with the leaf rust reaction of selected families and their resulting bulks were identified in each population.

### Genotyping polymorphic SNPs for F_8_ RILs using KASP and Fluidigm assays

Genomic DNA was extracted from the four F_8_ RIL populations as well as the parental lines, according to CIMMYT’s automated DNA extraction protocol, using a BIOMEK FX^p^ liquid handling station and the Sbeadex mini plant kit from LGC Genomics (LGC, Teddington, Middlesex, UK)[39]. After DNA quantification and quality assessment, all samples were diluted to approximately 50 ng/μl. Based on the results from the BSA done in the F_3_ generation, and the source sequence from which the 90K iSelect probes were originally developed, polymorphic SNPs identified within the candidate regions for leaf rust resistance were converted into KASP markers. For each SNP, two allele-specific forward primers and one common reverse primer were designed using the Primer3 software [40]. Seven additional publicly available KASP primers that were designed at the University of Bristol (http://www.cerealsdb.uk.net) were also used for genotyping. Primer sets of all 66 KASP markers used in the mapping of leaf rust resistance in Amria, Byblos, Geromtel_3 and Tunsyr_2 are listed in S1 Table.

KASP genotyping was performed for the Amria/ATRED #2 and Byblos/ATRED #2 RIL populations, according to the guidelines in the KBIOscience KASP SNP genotyping manual (http://www.kbioscience.co.uk/). Reactions were performed in 384 well plates, with a final reaction volume of 8 μl, which contained 2.5 μl of KASP 2X reaction mix, 50 ng of template DNA, 0.165 μM Hex forward primer, 0.165 μM FAM forward primer and 0.412 μM universal reverse primer. The following cycling conditions were used: 15 min at 94°C followed by 10 touchdown cycles of 20 s at 94°C and 60 s at 61°C (dropping 0.8°C per cycle); after the final annealing temperature of 57°C was achieved, there were 26 cycles of 20 s at 94°C and 60 s at 57°C, with a final fluorescence plate reading taken at 10°C. Thermocycling and fluorescence readings were performed on a Bio-Rad C1000 thermocycler and the data were analyzed using Bio-Rad CFX Manager software (Bio-Rad Laboratories Ltd, Hercules, CA, USA).

Because of the large number of markers to be genotyped for the populations derived from the crosses Geromtel_3/ATRED #2 and Tunsyr_2/ATRED #2 (55 and 35 markers, respectively), a high-throughput SNP genotyping platform was selected. The Fluidigm 192.24 Dynamic Array IFC (Integrated Fluidic Circuit) (Fluidigm Corp., South San Francisco, CA, USA) provides a solution for targeted high sample throughput SNP genotyping. It is designed to genotype 192 samples against 24 assays in a single run. Genotyping was carried out on the parental lines Geromtel_3, Tunsyr_2, ATRED #2 and the F_8_ RILs from both populations, following the procedures detailed in the manufacturer’s SNP genotyping analysis user guide (https://www.fluidigm.com). Specific Target Amplification (STA) primers were designed for each SNP, and STA was performed for all genomic DNA samples. A 1:100 dilution of the STA products was then used for sample mix preparation. The assay mix and sample mix were then loaded onto a 192.24 dynamic array chip, mixed and thermal-cycled using an IFC Controller HX and FC1 thermal cycler (Fluidigm Corp., South San Francisco, CA, USA), according to the manufacture’s protocols. End-point fluorescent images of the chip were acquired on an EP-1 imager, and the data was analyzed with the Fluidigm SNP Genotyping Analysis software (Fluidigm Corp., South San Francisco, CA, USA).

### Linkage mapping

SNP markers that showed a good quality of allele calling, based on the clusters of the scatter plots, were used for linkage analysis. Linkage maps of chromosome arms that carried the *Lr* genes were constructed using MapDisto 1.7.7 software [41], at a minimum logarithm of odds (LOD) score of 3 and maximum recombination fraction of 0.3. Co-segregating markers were identified in each population, and the marker with the lowest percentage of missing data was chosen to represent each cluster. Double recombinants were corrected using the functions ‘Show double recombinants,’ ‘Show error candidates’ and ‘Replace error candidates by flanking genotype’ as implemented in the MapDisto software [42]. The Kosambi function was used to convert the recombination fractions to centimorgans (cM) [42]. The final linkage maps were prepared using MapChart software [43].

### Genotyping simple sequence repeat (SSR) markers

Two SSR markers, *Xgwm344-7B* and *Xgwm146-7B*, which were previously shown to be linked to *Lr14a* [18] were used to screen the two resistant parents Amria and Byblos, as well as the susceptible parent ATRED #2. Subsets of resistant and susceptible RILs from both populations were also included. The French cultivar Sachem, previously reported to carry *Lr14a* [44], was included as a positive check. The same lines were also tested using four combinations of nucleotide-binding site leucine-rich repeat (NBS-LRR)-specific primers previously determined to be linked to *Lr14a* in a segregating population (*4406F*: CACGACGTTGTAAAACGACTTTCATTTTGTTCTCTCAGCCATA; *4407F*: CACGACGTTGTAAAACGACTTCATTTTGTTCTCTCAGCCATAC; *4840R*: GATGGATGATTTGGGTTTTTCTAC and *4852R*: TTACATGTGGATGATGGATGATTT) (C. Pozniak, *unpublished data*). The SSR marker *Xwmc487*, previously reported to be linked to *Lr61* on chromosome arm 6BS [19], was also used to genotype Geromtel_3 and Tunsyr_2. The durum wheat cultivar Guayacan INIA was used as a positive control that carries *Lr61*. The primer sequences of these SSR markers were obtained from the GrainGenes database (http://wheat.pw.usda.gov/GG3). PCR reactions were performed in 96 well plates with total reaction volumes of 25 μl, according to the protocols described by Herrera-Foessel et al. [19] and Pozniak et al. [45]. Dye-labeled M13 primer was added to the PCR mix for *Xgwm344-7B* and *Xgwm146-7B*; this allowed polymorphisms to be resolved using capillary electrophoresis on an ABI3130 genetic analyzer (Applied Biosystems^®^, Foster City, CA, USA). Polymorphisms for the NBS-LRR-specific primers and the SSR marker *Xwmc487* were scored on 2% agarose gels.

### Physical mapping of the polymorphic SNPs

The probe source sequences of all the SNP markers used in the final mapping of the *Lr* genes from all sources of resistance were physically mapped against the reference sequence of WEW accession ‘Zavitan’ [46] using GMAP [47]. The corresponding physical intervals for SNPs associated with leaf rust resistance in each population were identified under stringent parameters of coverage > 90% and identity > 95%. Genes falling within these physical intervals were identified using the available annotations for the WEW genome [46].

## Results

### Genetic characterization of the leaf rust resistance

The observed phenotypic distributions of host reactions at the F_3_, F_6_ and F_8_ generations supported segregation of a single dominant gene for leaf rust resistance in Amria, Geromtel_3 and Tunsyr_2 (Table 1). However, only the F_3_ and F_6_ RILs from the cross Byblos/ATRED#2 fit the ratios expected for segregation of a single gene (Table 1). The F_8_ RILs from the latter cross did, however, fit a ratio of 9:7, expected for resistance controlled by two complementary genes (*p* = 0.665).

**Table 1:** Classification of field reactions to the race BBG/BP of *P*. *triticina* of F_3_, F_6_ and F_8_ progenies from four crosses involving four sources of resistance crossed to the susceptible genotype ATRED #2.

Cross	F_2:3_ Families			F_2:6_ Families			F_8_ RILs		
	Hr:Seg:Hs	Ratio	*P*	Hr:Seg:Hs	Ratio (%)	*P*	R:S	Ratio	*P*
Amria/ATRED #2	48:123:48	1:2:1	0.189	121:3:92	48.5:3:48.5	0.065	113:100	1:1	0.411
Byblos/ATRED #2	51:120:60	1:2:1	0.591	105:5:114	48.5:3:48.5	0.618	128:93	1:1	0.046*
Geromtel_3/ATRED #2	46:98:35	1:2:1	0.227	107:8:76	48.5:3:48.5	0.052	102:87	1:1	0.309
Tunsyr_2/ATRED #2	43:108:52	1:2:1	0.443	97:12:97	48.5:3:48.5	0.084	103:101	1:1	0.944

F_2:3_ and F_2:6_ families were classified as homozygous resistant (Hr); segregating (Seg); and homozygous susceptible (Hs), based on the host reactions of the plants within each family. The F_8_ RILs host reactions were scored as resistant (R) or susceptible (S). The level of significance for segregation ratios determined by χ^2^ tests are indicated by *P*, *p*-value. The null hypothesis for the χ^2^ test was rejected at *p*-value < 0.05.

*p-value < 0.05 indicating that the observed segregation ratio is significantly different from the expected segregation ratio at a 95% level of confidence.

**Fig 1:**
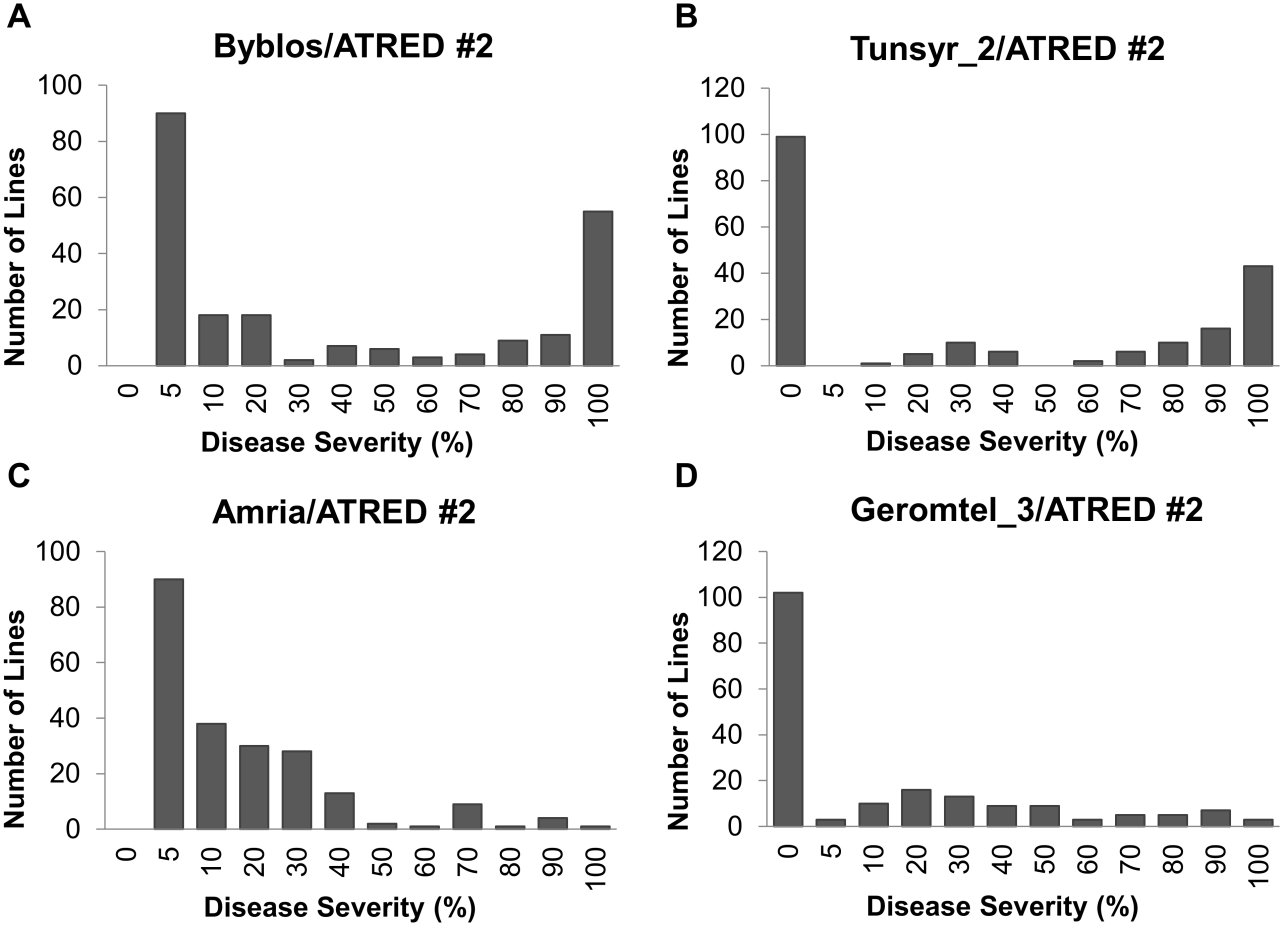
Frequency distributions of the disease severity (DS) scores in the F_6_ generation of four RIL populations. (A) Frequency distribution of DS for the Byblos/ATRED #2 population. (B) Frequency distribution of DS for the Tunsyr_2/ATRED #2 population. (C) Frequency distribution of DS for the Amria/ATRED #2 population. (D) Frequency distribution of DS for the Geromtel_3/ATRED #2 population.

The frequency distributions of the disease severity (DS) scores for the four F_6_ populations are represented in Fig 1. The four resistant parents showed the lowest DS scores (0–5%), and the highest scores (90–100%) were observed for the susceptible parent ATRED #2. Histograms of the DS recorded for Byblos/ATRED #2 (Fig 1A) and Tunsyr_2/ATRED #2 (Fig 1B) F_6_ populations revealed bimodal distributions, which is typical of traits under control of a major genetic factor. However, the DS data from Amria/ATRED #2 (Fig 1C) and Geromtel_3/ATRED #2 (Fig 1D) showed a skewed distribution towards increased resistance.

### Allelism tests

Allelism tests were performed to determine if the sources of resistance used in this study carried genes that were either allelic to each other or tightly linked. The absence of susceptible F_2_ plants from the cross involving Amria and Byblos suggests that these two genotypes may be carrying allelic or closely linked genes for leaf rust resistance (Table 2). The presence of susceptible plants in all the other crosses involving Amria indicates that the resistance in this genotype is different from those in Geromtel_3 and Tunsyr_2, and is neither allelic nor it is linked to *Lr61*, carried by the line Sooty_9/Rascon_37//Guayacan INIA. No susceptible plants were identified in all the crosses involving Geromtel_3, Tunsyr_2 and the *Lr61*-carrying Sooty_9/Rascon_37//Guayacan INIA, which suggested that the leaf rust resistance genes present in these three genotypes are either allelic or closely linked to each other. Altogether, these results indicate that Geromtel_3 and Tunsyr_2 carry genes that are the same, allelic, or tightly linked to *Lr61* and that Amria and Byblos share a separate resistance locus independent of *Lr61*.

**Table 2:** Number of resistant and susceptible F_2_ plants from crosses between different sources of resistance to leaf rust used for allelism testing.

Cross	Total F_2_ plants	Resistant	Susceptible
Amria/Byblos	200	200	0
Amria/Geromtel_3	250	220	30
Amria/Tunsyr_2	304	250	54
Geromtel_3/Tunsyr_2	275	275	0
Amria/Sooty_9/Rascon_37//Guayacan INIA	310	223	87
Byblos/Sooty_9/Rascon_37//Guayacan INIA	280	173	107
Geromtel_3/Sooty_9/Rascon_37//Guayacan INIA	301	301	0
Tunsyr_2/Sooty_9/Rascon_37//Guayacan INIA	276	276	0

### Linkage mapping

#### Leaf rust resistance in Amria and Byblos

**Fig 2:**
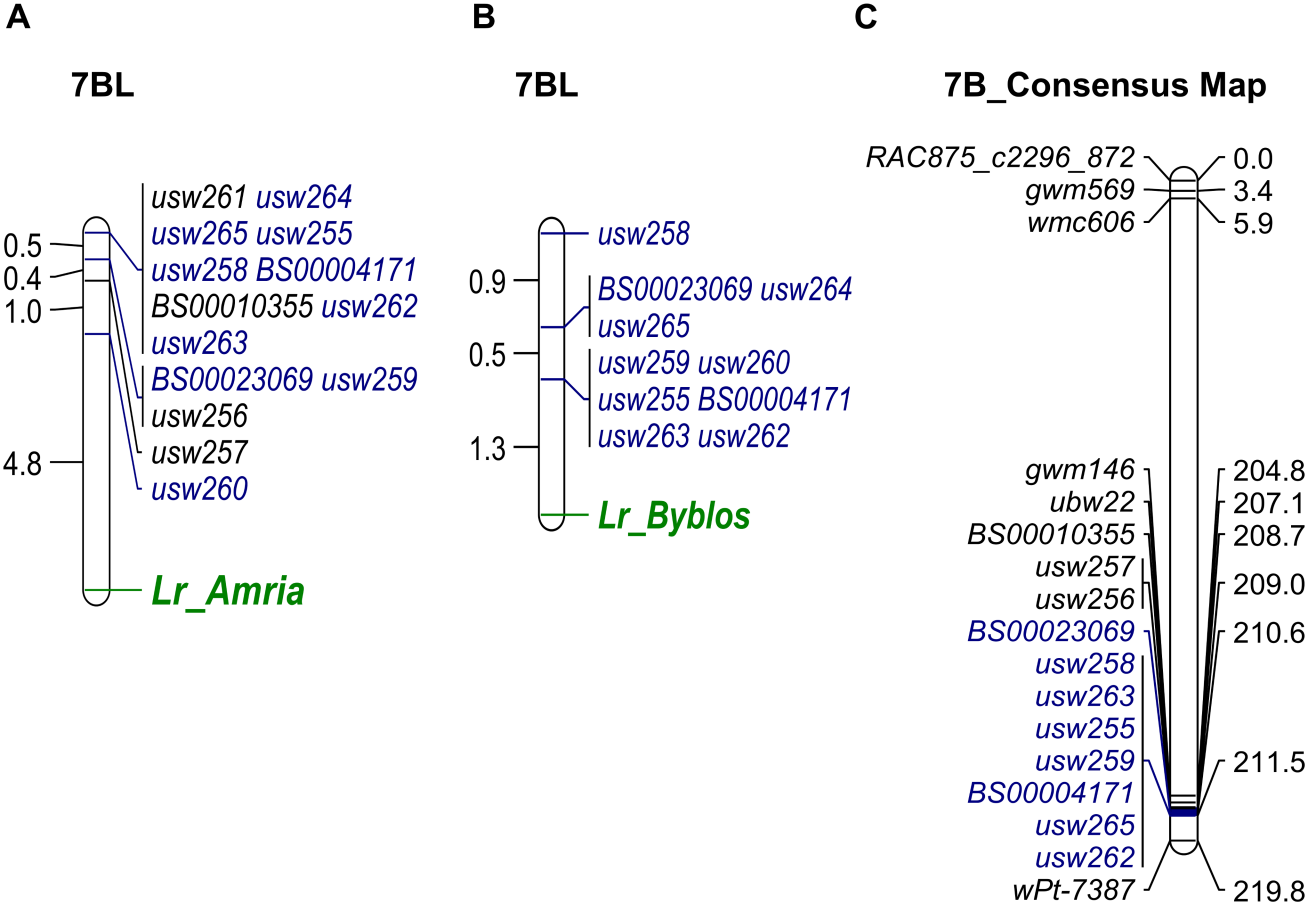
Linkage groups of KASP markers associated with the leaf rust resistance genes *Lr_Amria* and *Lr_Byblos* and their positions on the consensus map. (A) Markers associated with resistance in Amria. (B) Markers associated with resistance in Byblos. (C) High-density tetraploid consensus map for chromosome 7B [48]. Markers highlighted in blue are linked to the resistance in both Amria and Byblos. Genetic distances are displayed in cM.

BSA identified 28 and 24 SNPs that were genetically linked to the leaf rust resistance from Amria and Byblos, respectively; 22 of them were common between the two populations, supporting the allelism test results that indicated that Amria and Byblos may carry the same gene, alleles, or closely linked genes for leaf rust resistance. Based on the high-density consensus map of tetraploid wheat [48], a candidate region for leaf rust resistance in both genotypes was identified on the long arm of chromosome 7B. A list of all the SNP markers linked to the resistance in Amria and Byblos and their position on the consensus map is presented in S2 Table. Corresponding sequences from the Infinium assay were used to develop allele-specific KASP primers for 19 SNPs on chromosome 7BL, which were later tested on the parental lines. Only the markers that produced clear clusters for accurate genotype assignment were assayed on the entire RIL populations (S1 Table). Among the 19 KASP markers developed, only 14 and 10 were considered reliable for mapping in Amria/ATRED #2 and Byblos/ATRED #2 populations, respectively (Fig 2). All 14 markers that were closely linked to the leaf rust resistance gene in Amria (referred to as *Lr_Amria*) mapped within a 6.7 cM interval from the gene (Fig 2A). The KASP marker *usw260* was the closest to *Lr_Amria*, mapping at 4.8 cM proximal to the gene. In the Byblos/ATRED #2 population, 10 KASP markers mapped within 2.7 cM of the resistance gene in Byblos (Fig 2B). *Lr_Byblos* was located at 1.3 cM from the co-segregating markers *usw259*, *usw262*, *usw255*, *usw260*, *usw263* and *BS00004171*. All of these markers from both populations, mapped within an interval of 3 cM, spanning positions 208.7–211.5 cM on the tetraploid wheat consensus map (Fig 2C), providing compelling map-based evidence supporting the allelism or tight linkage between the resistance genes from both sources.

The distal region of chromosome 7B is known to carry the major leaf rust resistance gene *Lr14a*. Several markers reported to be linked to *Lr14a* were used to genotype the parental lines Amria, Byblos, ATRED #2, as well as resistant (R) and susceptible (S) RILs from the two mapping populations. The *Lr14a*-carrier “Sachem” was used as a positive control [44]. All lines, including the parents, showed polymorphism for both markers *Xgwm344* (S1 Fig) and *Xgwm146* (S2 Fig), when compared to Sachem. Furthermore, capillary electrophoresis analysis revealed different amplicon sizes for the parental lines Amria and Byblos compared to Sachem, for both markers (S1 and S2 Figs). For the *Xgwm344* marker, a single 152 base pairs (bp) DNA fragment was amplified for each of the parental lines Amria, Byblos and ATRED #2, clearly different from the 122 bp DNA fragment amplified for Sachem (S1 Fig). For the *Xgwm146* marker, two different amplicons were amplified for Sachem (174 bp and 189 bp) whereas both Amria and ATRED #2 were characterized by two amplicons of 172 bp and 206 bp, and Byblos had a single 179 bp fragment amplified (S2 Fig). Different combinations of NBS-LRR primers were used to screen the parental lines and the *Lr14a*-carrying Sachem, which were able to show either presence or absence of the NBS-LRR associated with *Lr14a* (S3 Fig). Only a single fragment was amplified from Sachem, indicating the presence of *Lr14a* in this cultivar; however, no PCR products were observed for any of the other lines tested. Altogether, our current data suggests that the major *Lr* gene present in Amria and Byblos is likely different from *Lr14a*.

#### Leaf rust resistance in Geromtel_3 and Tunsyr_2

BSA revealed that 115 SNPs were linked to the leaf rust resistance in Geromtel_3 and 67 SNPs were associated with the resistance in Tunsyr_2, including 52 common SNPs between the two sources of resistance. Based on the tetraploid wheat consensus map [48], a candidate region for the leaf rust resistance carried by both sources was identified on the short arm of chromosome 6B. A summary of all the SNP markers associated with the leaf rust resistance in Geromtel_3 and Tunsyr_2, and their positions on the consensus map are presented in S3 Table. Allele-specific KASP primers were developed for 56 SNPs on chromosome 6BS, which were used to genotype the parental lines as well as selected resistant and susceptible lines from each population. A total of 40 KASP markers that produced clear clusters were assayed on the Geromtel_3/ATRED #2 F_8_ RILs, whereas, only 28 markers were retained to genotype the Tunsyr_2/ATRED #2 RIL population. Primer sets of all KASP markers linked to leaf rust resistance in Geromtel_3 and Tunsyr_2 are listed in S1 Table.

**Fig 3:**
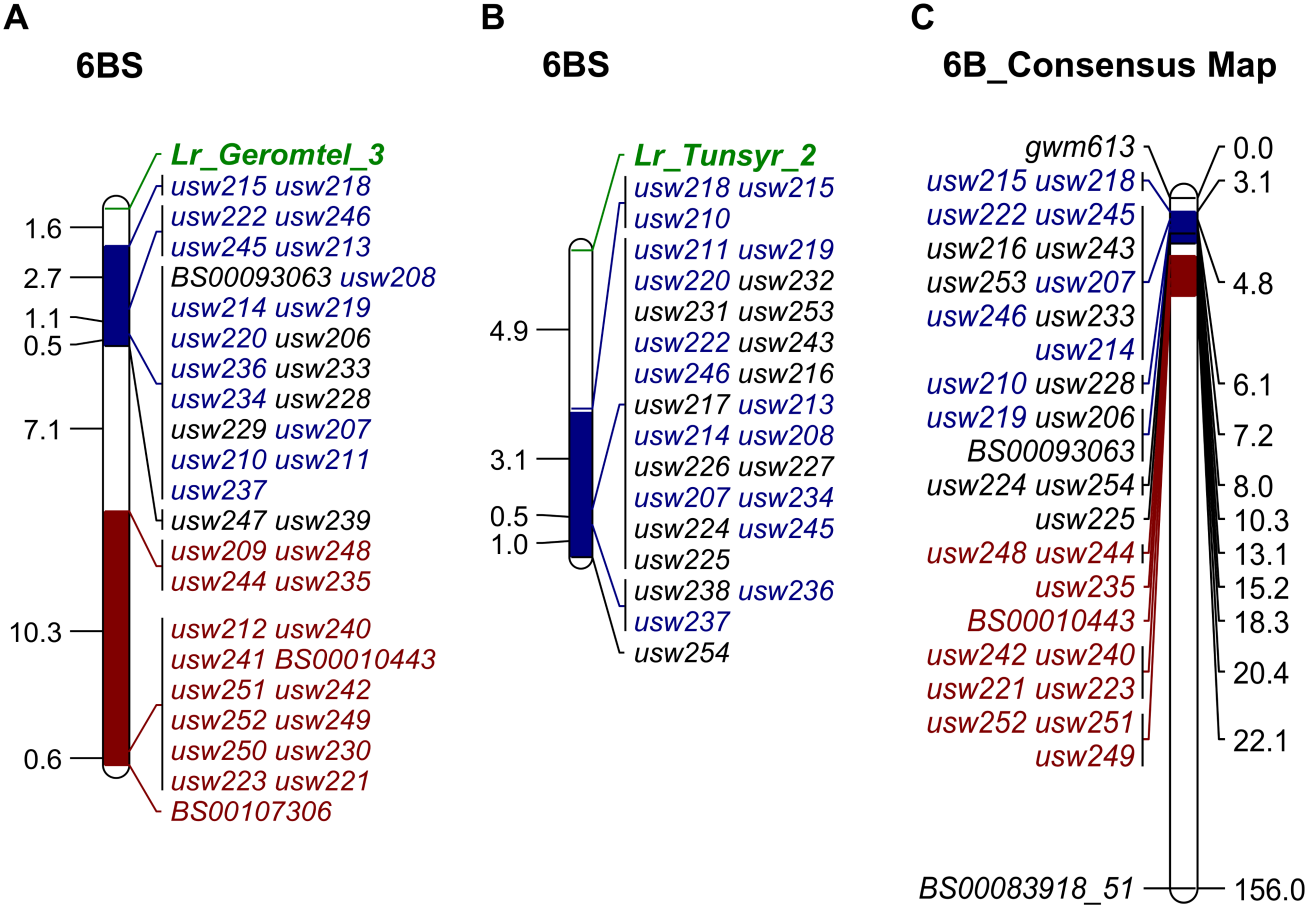
Linkage groups of KASP markers associated with the leaf rust resistance genes *Lr_Geromtel_3* and *Lr_Tunsyr_2* and their positions on the consensus map. (A) Markers associated with resistance in Geromtel_3. (B) Markers associated with resistance in Tunsyr_2. (C) High-density tetraploid consensus map for chromosome 6B [48]. Markers highlighted in blue are linked to the resistance in both Geromtel_3 and Tunsyr_2. Markers highlighted in red are linked only to the resistance in Geromtel_3. Genetic distances are displayed in cM.

The genetic map for the leaf rust resistance gene in Geromtel_3 (*Lr_Geromtel_3*) spanned an interval of approximately 23.9 cM (Fig 3A), with markers *usw215* and *usw218* mapping 1.6 cM from the gene and *usw222*, *usw245*, *usw213* and *usw246* mapping 4.3 cM from it. Fifteen other SNP markers mapped at approximately 5.4 cM from *Lr_Geromtel_3*. Another set of 17 markers (highlighted in red in Fig 3A) were linked exclusively to *Lr_Geromtel*_3, mapping between 13 to 23.9 cM distal to the gene, but were monomorphic in the Tunsyr_2 progenies.

A total of 28 markers were used to construct the genetic map for the leaf rust resistance locus in Tunsyr_2 (*Lr_Tunsyr_2*), which spanned a 9.5 cM interval (Fig 3B). Markers *usw210*, *usw215* and *usw218* mapped at 4.9 cM distal to *Lr_Tunsyr_2*.

While markers *usw215* and *usw218* were the closest to both *Lr_Geromtel_3* and *Lr_Tunsyr_2* (Fig 3A and 3B), only 16 markers were linked to the resistance in both populations (markers highlighted in blue in Fig 3). Twenty-four markers were exclusively associated to the resistance in Geromtel_3 and 12 markers were only linked to the resistance in Tunsyr_2.

The leaf rust resistance gene *Lr61* was identified in the Chilean durum wheat cultivar Guayacan INIA, and was mapped to the short arm of chromosome 6B, at approximately 28 cM from the SSR marker *Xwmc487* [19]. PCR amplicons for the marker *Xwmc487* were generated for the parental lines Geromtel_3, Tunsyr_2 and ATRED #2, as well as the cultivar Guayacan INIA (S4 Fig). The agarose gel revealed polymorphism between Guayacan INIA and the parental lines Geromtel_3 and ATRED #2, suggesting that *Lr61* may not be present in these genotypes. In this case, the results from the allelism tests would more likely indicate close linkage between the two genes (*Lr_Geromtel_3* and *Lr61*) rather than allelic relationship. Alternatively, the large distance between *Xwmc487* and *Lr61* could make this marker non-diagnostic for the presence of *Lr61* in genetic backgrounds that are different from the one that was used for its mapping. On the other hand, the PCR product amplified for Tunsyr_2 was similar to that amplified in Guayacan INIA, hampering our ability to determine whether the resistance from these two genotypes was due to the same gene or to different but closely linked genes.

### Physical mapping

DNA sequences associated with 10 SNP markers linked to the resistance in Amria and Byblos were positioned on the “Zavitan” reference sequence of tetraploid wheat (Table 3). Except for the marker *Ku_c6566_3086*, all SNP markers spanned a physical interval of about 6.7 megabase pairs (Mb) (746,587,151–753,310,876 bp). Five markers, namely *Tdurum_contig62213_423*, *tplb0045c05_154*, *BS00023069_51*, *BS00064933_51* and *Kukri_c20875_997*, were linked to the resistance in both Amria and Byblos and mapped within a 42,778 bp interval (747,105,190–747,147,968 bp). Likewise, sequences of 27 SNPs associated with the resistance in Geromtel_3 and/or Tunsyr_2 were physically mapped on the WEW genome (Table 4). All of the SNP markers linked to the resistance in Tunsyr_2 mapped within a 4Mb interval (5,812,642–9,797,172 bp). However, SNP markers linked to the resistance in Geromtel_3 spanned an interval of about 17.3 Mb (5,812,642–23,093,553 bp).

**Table 3:** Map positions of the SNP markers linked to leaf rust resistance in Amria and Byblos and their corresponding physical intervals in the WEW sequence of chromosome 7B.

SNP marker	KASP marker	Linkage to resistance^a^	Position Chr. 7B^b^	Position in WEW
*Ku_c6566_3086*	*usw260*	A and B	N/A	653,676,250
*Tdurum_contig30909_76*	*usw258*	A and B	211.5	746,587,151
*Tdurum_contig62213_423*	*usw264*	A and B	N/A	747,105,190
*tplb0045c05_154*	*usw265*	A and B	211.5	747,108,023
*BS00023069_51*	*BS00023069*	A and B	210.6	747,110,507
*BS00064933_51*	*usw255*	A and B	211.5	747,145,702
*Kukri_c20875_997*	*usw262*	A and B	211.5	747,147,870
*RAC875_c525_1372*	*usw257*	A	209.0	751,585,860
*BS00010355_51*	*BS00010355*	A	208.7	751,588,580
*Kukri_c17115_372*	*usw261*	A	N/A	753,310,785

^a^ A, Amria; B, Byblos.

^b^ Chr, chromosome; N/A, SNP map position not available from the consensus map.

**Table 4:** Map positions of the SNP markers linked to leaf rust resistance in Geromtel_3 and Tunsyr_2 and their corresponding physical intervals in the WEW sequence of chromosome 6B.

SNP marker	KASP marker	Linkage to resistance^a^	Position Chr. 6B^b^	Position in WEW
*Tdurum_contig43538_1687*	*usw246*	G and T	4.8	5,812,642
*Tdurum_contig43538_1582*	*usw245*	G and T	4.8	5,812,747
*Excalibur_c96134_182*	*usw222*	G and T	4.8	5,812,813
*CAP7_rep_c6852_87*	*usw216*; *usw217*	T	4.8	5,814,978
*BobWhite_c39821_195*	*usw213*	G and T	N/A	5,821,656
*BS00093063_51*	*BS00093063*	G	7.2	8,254,653
*RAC875_c31381_820*	*usw237*	G and T	7.2	8,438,520
*RAC875_c31381_883*	*usw238*	T	N/A	8,438,666
*Excalibur_c31801_48*	*usw219; usw220*	G and T	7.2	9,232,277
*IACX9205*	*usw224*	T	8	9,506,064
*Tdurum_contig52819_287*	*usw247*	G	N/A	9,546,765
*RAC875_c33407_350*	*usw239*	G	N/A	9,546,765
*RAC875_c1305_120*	*usw254*	T	8	9,797,072
*Wsnp_CD453605B_Ta_2_1*	*usw248*	G	13.1	12,461,455
*Tdurum_contig42655_1727*	*usw244*	G	13.1	12,470,253
*RAC875_c18689_1950*	*usw235*	G	15.2	15,305,559
*BS00010443_51*	*BS00010443*	G	18.3	18,454,200
*BobWhite_c34318_375*	*usw212*	G	N/A	19,978,536
*Kukri_c24795_267*	*usw230*	G	N/A	19,978,605
*RAC875_c38592_187*	*usw240; usw241*	G	20.4	19,983,498
*Excalibur_rep_c114123_366*	*usw223*	G	20.4	19,983,705
*Excalibur_c64989_556*	*usw221*	G	20.4	19,983,996
*RAC875_rep_c105906_124*	*usw242*	G	20.4	19,984,203
*Wsnp_Ex_c702_1383612*	*usw252*	G	22.1	21,730,858
*wsnp_Ex_c702_1382859*	*usw251*	G	22.1	21,733,535
*Wsnp_Ex_c4728_8444212*	*usw249; usw250*	G	22.1	21,736,168
*BS00107306_51*	*BS00107306*	G	N/A	23,093,453

^a^ G, Geromtel_3; T, Tunsyr_2.

^b^Chr, chromosome; N/A, SNP map position not available from the consensus map.

Several transcripts coding for putative NBS-LRR proteins, resistance gene analogues (RGA2), RPM1 and RPP13-like disease resistance proteins, as well as proteases and ABC transporter were identified within the 7BL interval for the leaf rust resistance in Amria and Byblos (S4 Table). Several NBS-LRR-encoding sequences have also been identified in the interval for Geromtel_3 and Tunsyr_2 on chromosome 6BS (S5 Table). Other candidate genes for the leaf rust resistance in Geromtel_3 and Tunsyr_2 include a HR-like lesion-inducing protein as well as a zinc finger peptidase and sugar transporters. The marker *usw224* that was linked to the resistance in Tunsyr_2 was mapped within an RPP13-like disease resistance gene (S5 Table).

## Discussion

To sustain the economic viability of durum wheat production globally, it is necessary to protect crops from the potentially destructive impact of rusts, including leaf rust. This is most effectively done by identifying and deploying new sources of resistance that are able to durably mitigate the threat of a dynamic and rapidly evolving pathogen population. In an effort to identify new sources of resistance to leaf rust, CIMMYT selected to characterize four resistant genotypes, namely Amria, Byblos, Geromtel_3 and Tunsyr_2, for their all stage resistance to all Mexican and Mediterranean pathotypes of *P*. *triticina* [35]. Studies of the inheritance of the leaf rust resistance from these four sources suggested that they all carry major resistance genes. Allelism testing results suggested that Amria and Byblos may share the same or closely linked resistance genes. Likewise, the resistance genes in Geromtel_3 and Tunsyr_2 were shown to be either allelic or tightly linked to each other and to the previously designated gene *Lr61* [19]. Furthermore, pedigree analysis showed that the two ICARDA lines Geromtel_3 and Tunsyr_2 share a common parent, namely the Tunisian breeding line D68.1.93A.1A (M.S. Gharbi, *personal communication*), which may be the original source of their leaf rust resistance [35]. The hypotheses of allelic or tightly linked genes were confirmed by the results of the BSA approach that identified two genomic regions associated with leaf rust resistance in these genotypes; one on chromosome 7BL for the resistance in Amria and Byblos, and the other on chromosome 6BS for the resistance in Geromtel_3 and Tunsyr_2.

Several studies, including association mapping and QTL analyses, have reported the importance of the distal region of chromosome arm 7BL in wheat resistance to leaf rust [44,49,50]. Indeed, three leaf rust resistance genes (i.e. *Lr14a*, *Lr14b* and *Lr68*) have been located on chromosome 7BL [51]. *Lr68* is an adult-plant resistance gene, with a partial resistance effect that confers a slow-rusting phenotype, identified in the common wheat cultivar Parula and is flanked by markers *Psy-1-1* and *Xgwm146* [51]. In contrast, the resistance in Amria and Byblos is expressed at the seedling stage [35] and shows a more complete resistance than *Lr68*, making *Lr68* an impossible candidate for the resistance in these two sources. The race-specific resistance gene *Lr14b* is very closely linked to *Lr14a* [52]. However, virulence against *Lr14b* is very common among *P*. *triticina* races that infect durum wheat, including the Mexican race BBG/BP that was used in the present study [2,51]. Since Amria and Byblos were resistant to this race, it can be concluded that their *Lr* genes are distinct from *Lr14b*. The final candidate gene located on chromosome 7BL, *Lr14a*, is linked to SSR markers *Xgwm344-7B* and *Xgwm146-7B* [18]. According to studies conducted by CIMMYT on a wide range of germplasm groups worldwide, *Lr14a* is present in the great majority of the resistant durum genotypes, and could represent the most common source of leaf rust resistance currently exploited by durum wheat breeders, globally. The over-reliance on *Lr14a* is dangerous since this gene was overcome in several areas around the Mediterranean Basin, including France [13], Tunisia [53] and Spain [14]. The ITs of Amria and Byblos were contrastingly different from those of *Lr14a*-carrying durum wheat genotypes indicating that the former cultivars were not carrying the gene (K. Ammar, *unpublished data*). In addition, Goyeau et al. [54] investigated the structure and evolution of the French durum *P*. *triticina* population using a durum wheat differential set, including Byblos. The French commercial cultivar Byblos was the only genotype displaying low infection types to all French pathotypes, including a pathotype which was virulent for both *Lr14a* and *Lr14b* alleles. Furthermore, the ITs of Byblos and those of Thatcher isolines, which carry known resistance genes, were different, leading to the conclusion that Byblos carried unknown gene(s) for resistance. Finally, neither Amria nor Byblos were positive for the molecular markers known to be linked to *Lr14a*, including *Xgwm344* (S1 Fig), *Xgwm146* (S2 Fig) and the NBS-LRR-specific primers *4406F/4840R*,*4406F/4852R*,*4407F/4840R* and *4407F/4852R* (S3 Fig). Altogether, these results suggest that the major *Lr* gene present in Amria and Byblos is different from *Lr14a* and is likely to be previously uncharacterized leaf rust resistance gene, making these two cultivars good candidates for exploring alternative sources of resistance for durum rust breeding.

Recently, the leaf rust resistance gene *LrBi16* has been mapped on chromosome 7BL of the Chinese bread wheat cultivar Bimai 16, and was reported to be allelic to *Lr14a* [55]. Xing et al. [56] also identified *LrFun* on the long arm of chromosome 7B of the Romanian bread wheat line Fundulea 900 and mapped it at 4.4 cM from the SSR marker *Xgwm344-7B*. Additional studies will be required to determine the relationship between these resistance genes and the genes from Amria and Byblos.

Linkage analysis positioned the leaf rust resistance locus in Amria and Byblos at the distal end of chromosome 7BL. The KASP marker *usw260* was the most tightly linked marker to the resistance in both genotypes; however, the distance to the *Lr* gene varied from 1.3 cM in Byblos to 4.8 cM in Amria. The variation in the length of the *Lr_Amria*-*usw260* and *Lr_Byblos*-*usw260* intervals between mapping populations suggests that recombination rates in this region of chromosome 7BL may vary between crosses or that insertion/deletion occurred in Amria or Byblos in the interval harboring the gene and the *usw260* SNP marker. Since this variation in marker-gene genetic distances was also observed for other SNP markers such as *usw255*, *usw258*, *usw262*, *usw263*, *usw264*, *usw265*, *BS00023069* and *BS0004171*, *usw260* remains the best marker to use in breeding, given its tight linkage to the resistance in both sources.

Three leaf rust resistance genes have been reported to map to chromosome arm 6BS, namely, *Lr36*, *Lr53* and *Lr61*. Both *Lr36* and *Lr53* originate from wild grasses relatives. *Lr36* was derived from *T*. *speltoides* and backcrossed into hexaploid wheat [57]. Marais et al. [58] reported the introgression of *Lr53* from *T*. *dicoccoides* to the short arm of chromosome 6B in common wheat. However, no reports are available to indicate that either *Lr36* or *Lr53* have been transferred to durum wheat. Furthermore, pedigree information of Geromtel_3 and Tunsyr_2 do not indicate any relationship to any of the wild relatives carrying these genes [35], though, *Lr53* cannot be fully ruled out as a candidate, since *T*. *dicoccoides* (genome AABB) is the wild progenitor of durum wheat. Herrera-Foessel et al. [19] identified *Lr61* on chromosome arm 6BS in the durum wheat cultivar Guayacan INIA to be linked to the SSR marker *Xwmc487*, but at a rather large distance (28.5 cM). *Lr61* is a partially dominant gene [19], but phenotypic analyses of the F_1_ plants from the crosses Geromtel_3/ATRED #2 and Tunsyr_2/ATRED #2 suggested that these cultivars carry completely dominant genes for leaf rust resistance [35]. Genotyping with *Xwmc487* revealed polymorphism between the fragments amplified for Geromtel_3 compared to Guayacan INIA, but not in the case of Tunsyr_2, when compared to the same check (S4 Fig), which suggests that the resistance in Tunsyr_2 may be allelic to *Lr61*, whereas Geromtel_3 carries a potentially different but closely linked gene. However, these marker results cannot be considered conclusive given the large distance between *Xwmc487* and *Lr61*, and the absence of high-density maps for the original *Lr61* mapping population.

The distributions of markers linked to the leaf rust resistance in Geromtel_3 on both the consensus linkage map (Fig 3C) and the WEW pseudomolecules (S3 Table) suggest that Geromtel_3 may be carrying two different but tightly linked major *Lr* genes. One of these genes is likely allelic to *Lr61* and to *Lr_Tunsyr_2*, mapping to the distal end of chromosome 6BS, and the second is only present in Geromtel_3 and located centrally at about 17 Mb from the first *Lr* gene.

Physical mapping of the SNP markers linked to the resistance in Amria, Byblos, Geromtel_3 and Tunsyr_2 to the WEW reference sequence enabled the identification of candidate genes for leaf rust resistance, including NBS-LRR disease resistance proteins, RPP13-like and RPM1 disease resistance proteins, as well as several receptor kinases (S4 and S5 Tables). NBS-LRR proteins are the most abundant class of disease resistance genes in plants. This protein family includes two major subfamilies, based on the features of their N-terminal structures: the Toll-interleukin (TIR-NBS-LRR) subfamily, and the coiled-coil (CC-NBS-LRR) subfamily [59–61]. Leaf rust resistance genes *Lr21* [62], *Lr10* [63], and *Lr1* [64], stem rust resistance genes *Sr33* [65] and *Sr35* [66], and the powdery mildew resistance gene *Pm3b* [67], are six resistance genes that have been cloned in wheat. All six proteins contain CC, NBS and LRR motifs. TIR-NBS-LRR genes represent the majority of the *R* genes in *Arabidopsis*; however, disease resistance proteins with a TIR N-terminal domain have not yet been reported in cereals [59]. These abundant NBS-LRR disease resistance proteins act as immune receptors and are involved in the detection of diverse pathogens through direct or indirect perception of pathogen *Avr* proteins [61,68,69]. Pathogens are able to evade recognition and overcome plant resistance through *Avr* gene mutation. Hence, both pathogen and host plant undergo parallel molecular diversification to secure their survival, leading to the concept of evolutionary race between pathogen virulence and plant defense [60,69–71]. This plant-pathogen coevolution could explain the rapid breakdown of leaf rust resistance conferred by most seedling, race-specific *Lr* genes in wheat, especially when the same genes are deployed in many cultivars grown over large areas, allowing for the rapid adaptation and spread of new virulent races [71]. Several NBS-LRR proteins were identified within the physical intervals associated with the leaf rust resistance in Amria, Byblos, Geromtel_3 and Tunsyr_2 (S4 and S5 Tables). Resistance gene clusters have been reported in several plant genome studies and are the result of either segmental duplications that involve many genes, or ectopic duplications that move single genes or small gene clusters to unlinked loci [59–61]. Plant *R* genes are subject to several selective forces to cope with the rapidly evolving pathogen *Avr* genes. Alteration of *R* gene clusters, through diversification and gene conversion, results in increased variation and promotes the generation of novel resistance specificity [64]. Further investigations will be required to confirm the specific identity of the genes associated with leaf rust resistance in Amria, Byblos, Geromtel_3, and Tunsyr_2.

## Conclusions

Results from this study indicated that the durum wheat cultivars Amria and Byblos carry allelic or closely linked *Lr* genes on the long arm of chromosome 7B. Based on molecular marker analysis and previous genetic studies, it was concluded that none of these cultivars carried *Lr14a*, a widely-deployed resistance gene in durum wheat that is located on chromosome 7BL. Similarly, the leaf rust resistance in Geromtel_3 and Tunsyr_2 was mapped to chromosome 6BS. Allelism tests revealed that the genes in these lines are either allelic or closely linked to each other and to *Lr61*. Linkage map analysis in the genomic region responsible for this resistance suggested that Geromtel_3 may carry an additional gene, different from the one carried by Tunsyr_2.

Physical mapping identified several candidate genes for the leaf rust resistance in these lines that were mainly NBS-LRR proteins, which commonly act as *R* genes in plants. The results from the present study highlight the importance of chromosome arms 6BS and 7BL as regions rich in leaf rust resistance genes, which can be valuable in breeding programs for pyramiding multiple genes, to achieve more durable resistance. KASP markers tightly linked to these *Lr* genes have been produced and tested, and are ready to be used in applied breeding programs with high reliability and throughput.

## Supporting information

S1 Table KASP primer sets for the SNP markers associated with leaf rust resistance in Amria, Byblos, Geromtel_3 and Tunsyr_2. Markers starting with “*usw*” were designed by the durum wheat molecular lab at the University of Saskatchewan. Markers starting with “*BS*” were designed at the University of Bristol (http://www.cerealsdb.uk.net).
(XLSX)

S2 Table List of the SNP markers linked to leaf rust resistance in Amria and Byblos and their positions on the tetraploid wheat consensus map and in the wild emmer wheat (WEW) reference sequence. N/A, mapping information not available.
(XLSX)

S3 Table List of the SNP markers linked to leaf rust resistance in Geromtel_3 and Tunsyr_2 and their positions on the tetraploid wheat consensus map and in the wild emmer wheat (WEW) reference sequence. N/A, mapping information not available.
(XLSX)

S4 Table List of annotated genes within the *Lr_Amria* and *Lr_Byblos* intervals. (XLSX)

S5 Table List of annotated genes within the *Lr_Geromtel_3* and *Lr_Tunsyr_2* intervals. (XLSX)

S1 Fig PCR amplicons for the SSR marker *Xgwm344* linked to *Lr14a*. 1Kb+ DNA ladder; lanes 1–2, Amria; 3–4 resistant RILs from Amria/ATRED #2; 5–6 susceptible RILs from Amria/ATRED #2; 7–8 Byblos; 9–10 resistant RILs from Byblos/ATRED #2; 11–12 susceptible RILs from Byblos/ATRED #2; 13–14 Sachem (*Lr14a*+); and 15–16 ATRED #2.
(PDF)

S2 Fig PCR amplicons for the SSR marker *Xgwm146* linked to *Lr14a*. 1Kb+ DNA ladder; lanes 1–4 Amria; 5–8 Byblos; 9–10 Sachem *(Lr14a*+); 11–14 resistant RILs from Amria/ATRED #2; 15–18 resistant RILs from Byblos/ATRED #2; 19–22 susceptible RILs from Amria/ATRED #2; 23–26 susceptible RILs from Byblos/ATRED #2; 27–30 ATRED #2; and 31–32 Sachem (*Lr14a*+).
(PDF)

S3 Fig Banding pattern of the PCR products for the NBS-LRR-specific primers associated with *Lr14a*. (A) *4406F*/*4840R*. (B) *4406F*/*4852R*. (C) *4407F*/*4840R*. (D) *4407F*/*4852R*. 1Kb+, DNA ladder; Am, Amria; By, Byblos; AT, ATRED #2; Sa, Sachem *(Lr14a*+).
(PDF)

S4 Fig PCR amplicons for the SSR marker *Xwmc487* linked to *Lr61*. 1Kb+, DNA ladder; Gr_3, Geromtel_3; Tn_2, Tunsyr_2; G. INIA, Guayacan INIA (*Lr61*+); ATRED, ATRED #2.
(PDF)
