# Supplementary material for: Characterization and mapping of leaf rust resistance in four durum wheat cultivars
Source: PLoS One. 2018 May 10;13(5):e0197317. doi: 10.1371/journal.pone.0197317 (PMC5945016; doi:10.1371/journal.pone.0197317)
Supplement: S1 Fig — 1Kb+ DNA ladder; lanes 1–2, Amria; 3–4 resistant RILs from Amria/ATRED #2; 5–6 susceptible RILs from Amria/ATRED #2; 7–8 Byblos; 9–10 resistant RILs from Byblos/ATRED #2; 11–12 susceptible RILs from Byblos/ATRED #2; 13–14 Sachem (Lr14a+); and 15–16 ATRED #2. (PDF) [file pone.0197317.s006.pdf]

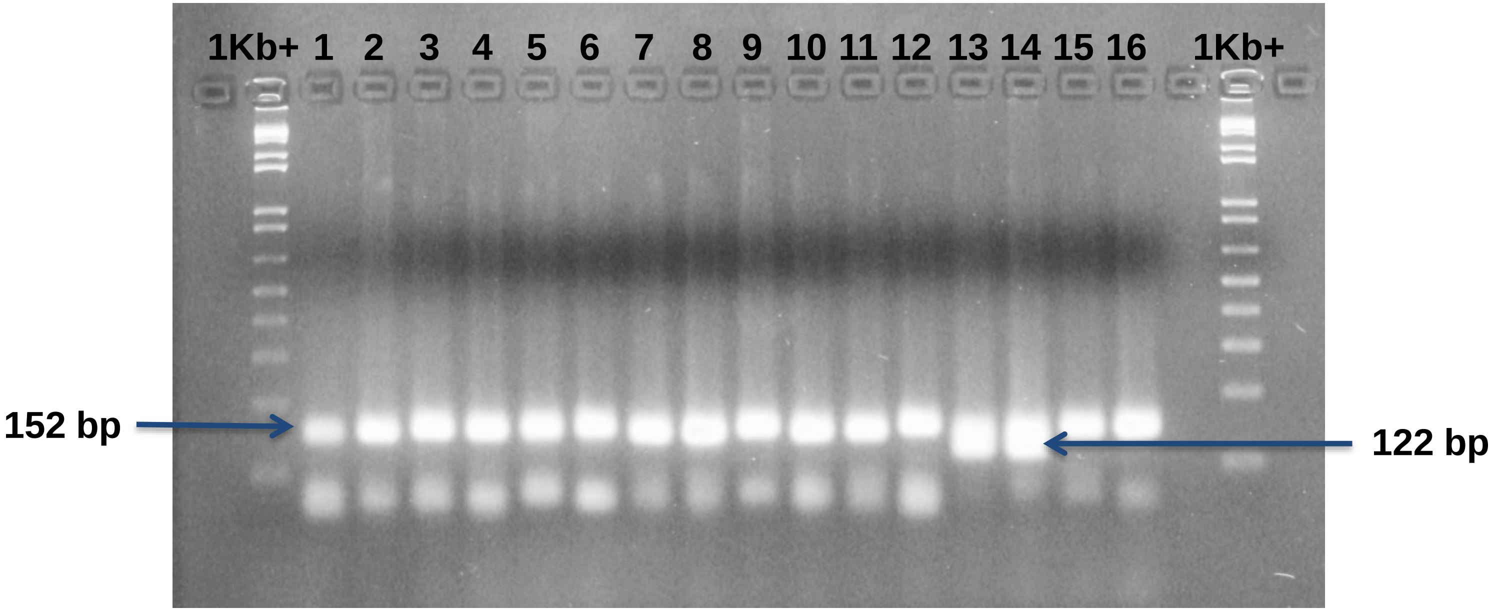

S1 Fig. PCR amplicons for the SSR marker *Xgwm344* linked to *Lr14a*. 1Kb+, DNA ladder; lanes 1-2, Amria; 3-4; resistant RILs from Amria/ATRED #2, 5-6; susceptible RILs from Amria/ATRED #2; 7-8, Byblos; 9-10, resistant RILs from Byblos/ATRED #2; 11-12, susceptible RILs from Byblos/ATRED #2; 13-14, Sachem (*Lr14a*+); and 15-16, ATRED #2.
