# Supplementary material for: Characterization and mapping of leaf rust resistance in four durum wheat cultivars
Source: PLoS One. 2018 May 10;13(5):e0197317. doi: 10.1371/journal.pone.0197317 (PMC5945016; doi:10.1371/journal.pone.0197317)
Supplement: S2 Fig — 1Kb+ DNA ladder; lanes 1–4 Amria; 5–8 Byblos; 9–10 Sachem (Lr14a+); 11–14 resistant RILs from Amria/ATRED #2; 15–18 resistant RILs from Byblos/ATRED #2; 19–22 susceptible RILs from Amria/ATRED #2; 23–26 susceptible RILs from Byblos/ATRED #2; 27–30 ATRED #2; and 31–32 Sachem (Lr14a+). (PDF) [file pone.0197317.s007.pdf]

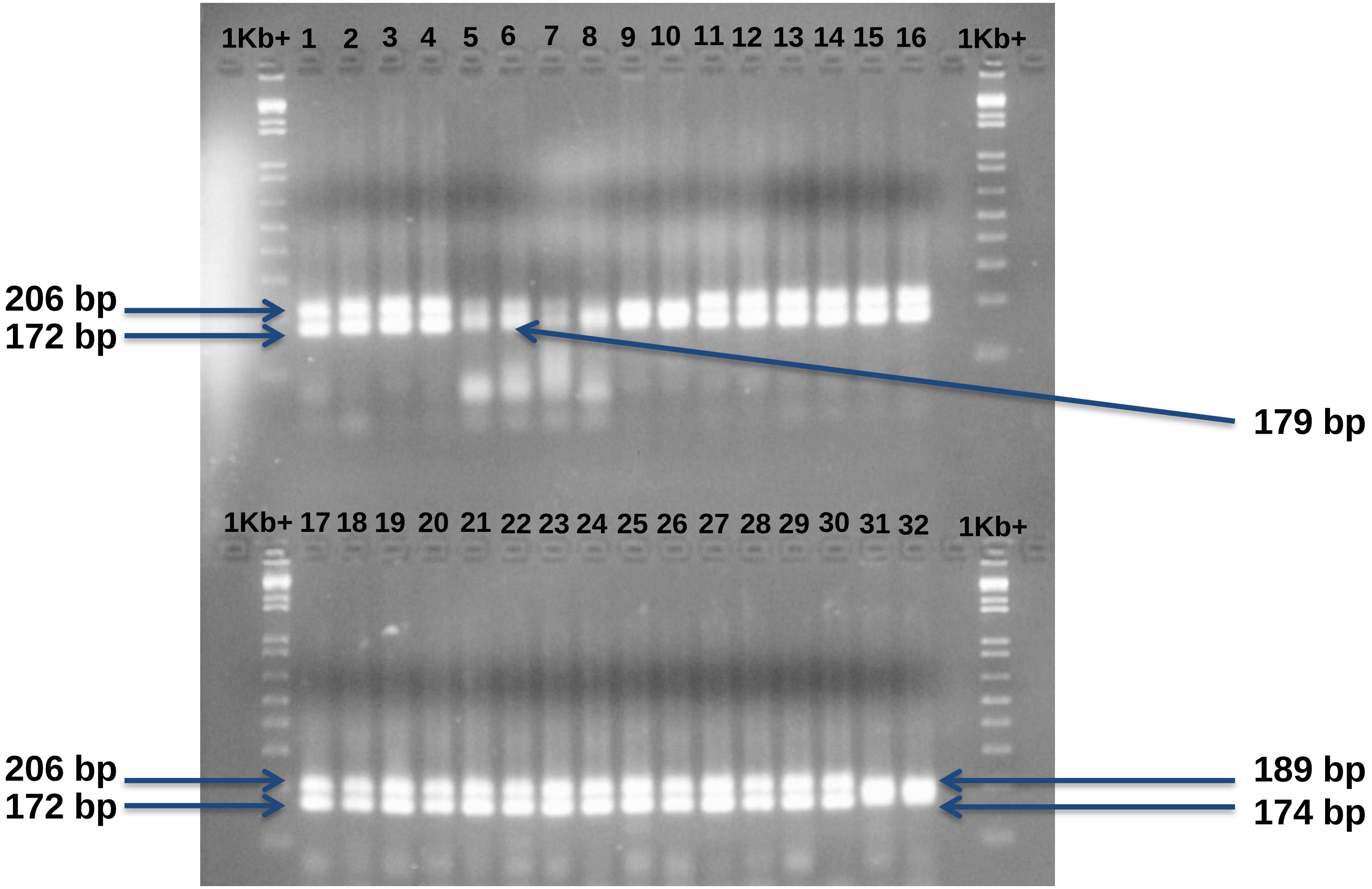

S2 Fig. PCR amplicons for the SSR marker *Xgwm146* linked to *Lr14a*. 1Kb+, DNA ladder; lanes 1-4, Amria; 5-8, Byblos; 9-10, Sachem (*Lr14a*+); 11-14; resistant RILs from Amria/ATRED #2; 15-18, resistant RILs from Byblos/ATRED #2; 19-22; susceptible RILs from Amria/ATRED #2; 23-26, susceptible RILs from Byblos/ATRED #2; 27-30, ATRED #2; and 31-32, Sachem (*Lr14a*+).
