# Supplementary material for: Characterization and mapping of leaf rust resistance in four durum wheat cultivars
Source: PLoS One. 2018 May 10;13(5):e0197317. doi: 10.1371/journal.pone.0197317 (PMC5945016; doi:10.1371/journal.pone.0197317)
Supplement: S3 Fig — (A) 4406F/4840R. (B) 4406F/4852R. (C) 4407F/4840R. (D) 4407F/4852R. 1Kb+, DNA ladder; Am, Amria; By, Byblos; AT, ATRED #2; Sa, Sachem (Lr14a+). (PDF) [file pone.0197317.s008.pdf]

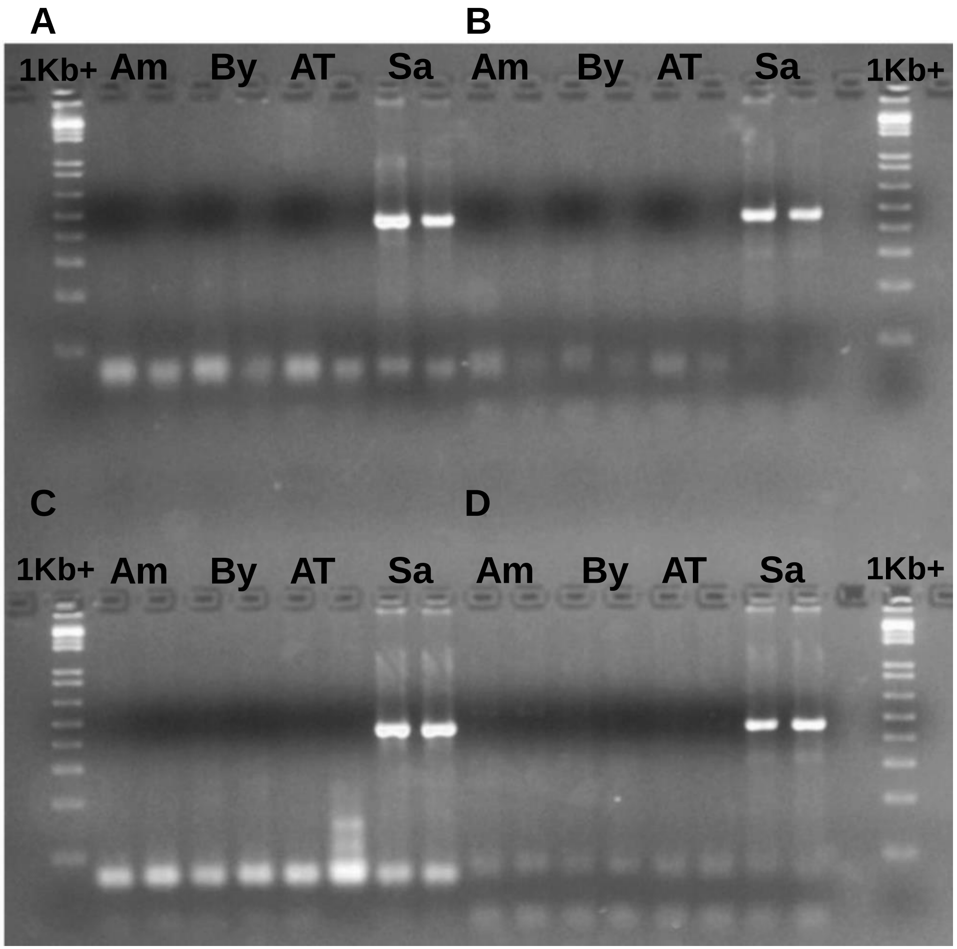

S3 Fig. Banding pattern of the PCR products for the NBS-LRR-specific primers associated with *Lr14a*. (A) *4406F/4840R*. (B) *4406F/4852R*. (C) *4407F/4840R*. (D) *4407F/4852R*. 1Kb+, DNA ladder; Am, Amria; By, Byblos; AT, ATRED #2; Sa, Sachem (*Lr14a*+).
