# Supplementary material for: Characterization and mapping of leaf rust resistance in four durum wheat cultivars
Source: PLoS One. 2018 May 10;13(5):e0197317. doi: 10.1371/journal.pone.0197317 (PMC5945016; doi:10.1371/journal.pone.0197317)
Supplement: S4 Fig — 1Kb+, DNA ladder; Gr_3, Geromtel_3; Tn_2, Tunsyr_2; G. INIA, Guayacan INIA (Lr61+); ATRED, ATRED #2. (PDF) [file pone.0197317.s009.pdf]

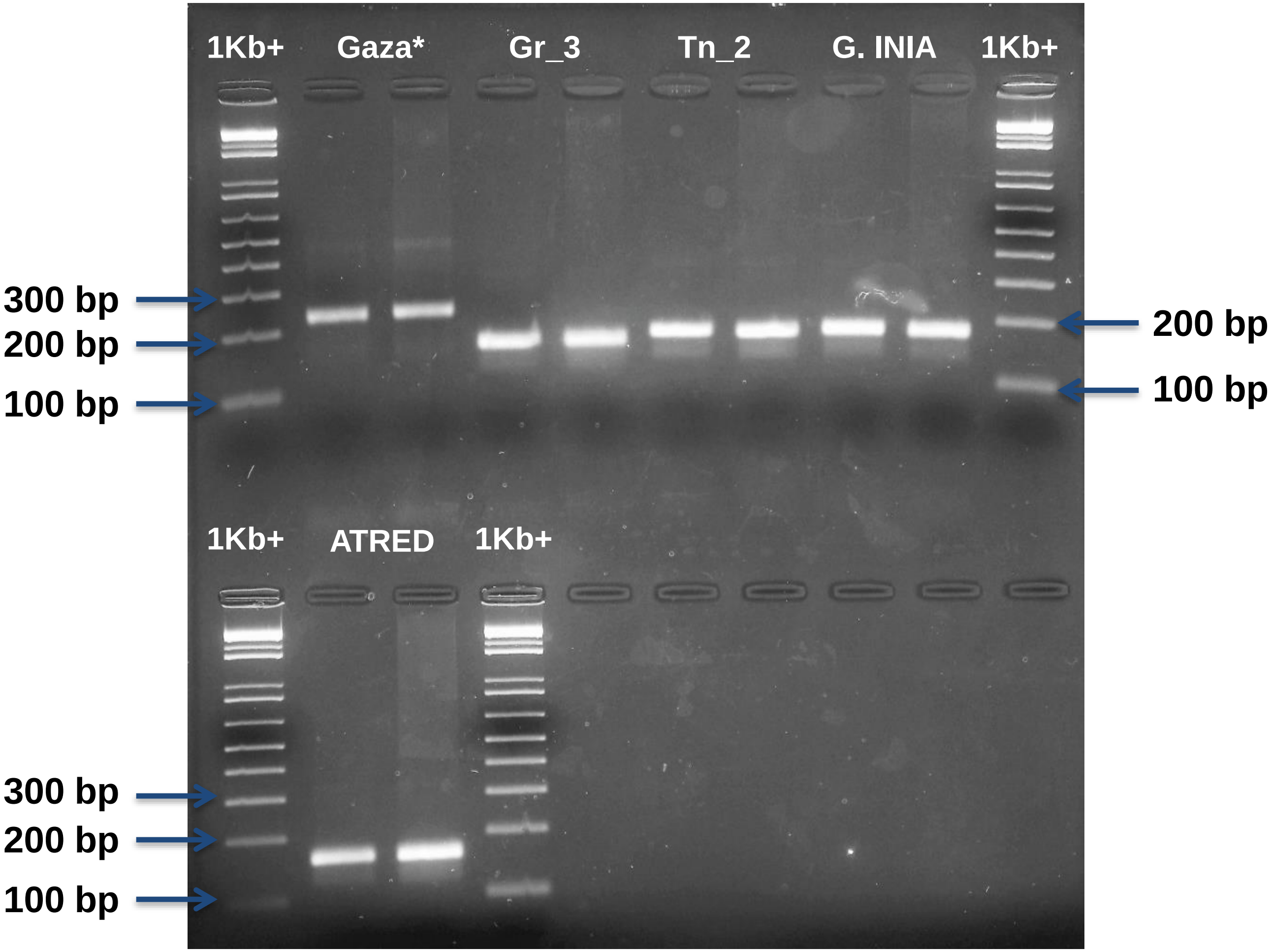

S4 Fig. PCR amplicons for the SSR marker *Xwmc487* linked to *Lr61*. 1Kb+, DNA ladder; Gr_3, Geromtel_3; Tn_2, Tunsyr_2; G. INIA, Guayacan INIA *(Lr61*+); ATRED, ATRED #2.

* Gaza is a durum wheat landrace carrying leaf rust resistance on chromosome 6BS (D. Kthiri, manuscript in preparation).
